# RNA polymerase I mutant affects ribosomal RNA processing and ribosomal DNA stability

**DOI:** 10.1080/15476286.2024.2381910

**Published:** 2024-07-24

**Authors:** Christophe Normand, Christophe Dez, Lise Dauban, Sophie Queille, Sarah Danché, Sarra Abderrahmane, Frederic Beckouet, Olivier Gadal

**Affiliations:** Molecular, Cellular and Developmental Biology Unit (MCD), Centre de Biologie Integrative (CBI), University of Toulouse, CNRS, UPS, Toulouse, France

**Keywords:** ribosomal RNA, Transcription, Genome stability, S.cerevisiae

## Abstract

Transcription is a major contributor to genomic instability. The ribosomal RNA (rDNA) gene locus consists of a head-to-tail repeat of the most actively transcribed genes in the genome. RNA polymerase I (RNAPI) is responsible for massive rRNA production, and nascent rRNA is co-transcriptionally assembled with early assembly factors in the yeast nucleolus. In *Saccharomyces cerevisiae*, a mutant form of RNAPI bearing a fusion of the transcription factor Rrn3 with RNAPI subunit Rpa43 (CARA-RNAPI) has been described previously. Here, we show that the CARA-RNAPI allele results in a novel type of rRNA processing defect, associated with rDNA genomic instability. A fraction of the 35S rRNA produced in CARA-RNAPI mutant escapes processing steps and accumulates. This accumulation is increased in mutants affecting exonucleolytic activities of the exosome complex. CARA-RNAPI is synthetic lethal with monopolin mutants that are known to affect the rDNA condensation. CARA-RNAPI strongly impacts rDNA organization and increases rDNA copy number variation. Reduced rDNA copy number suppresses lethality, suggesting that the chromosome segregation defect is caused by genomic rDNA instability. We conclude that a constitutive association of Rrn3 with transcribing RNAPI results in the accumulation of rRNAs that escape normal processing, impacting rDNA organization and affecting rDNA stability.

## Introduction

Genome integrity is essential for cell cycle survival. Cellular processes including DNA replication, repair, recombination and transcription are known to affect genomic stability [1,2]. Coordinated mechanisms have been selected to ensure genome integrity and cell proliferation. Ribosomal DNA genes (rDNA) are by far the most transcribed region of the genome, and are organized in large array of head-to-tail repeats, making this genomic locus potentially at risk during replication [3].

In *Saccharomyces cerevisiae*, budding yeast contains one rDNA locus on the right arm of chromosome XII, consisting of 100 to 200 repetitions. Each repeat unit comprises two highly transcribed elements, the 35S and 5S genes, respectively, transcribed by RNA polymerase I (RNAPI) and RNA polymerase III, that are flanked by two intergenic spacers: intergenic spacers (IGS) 1 and 2. Recent studies have shown that cells exploit genomic instability to achieve adaptation of rDNA copy number to environmental conditions [4]. The Fob1 protein is a multifunctional protein that binds to specific DNA sequences called RFB (Replication Fork Barrier) sites present in IGS1, creating a polar replication fork arrest [5,6]. Such replication fork barrier prevents collision between RNAPI transcription complexes and replication forks from the opposite direction. Counterintuitively, this stalled replication forks created by Fob1 promotes genomic instability through the displacement of cohesins [7,8]. Numerous mutants affecting rDNA homoeostasis at various level have been identified: they influence replication fork stalling, affect replication, interfere on IGS1 transcriptional activity or alter the loading rate and/or activities of condensin, cohesin and monopolin complexes on rDNA. In most of these mutants, the invalidation of Fob1 suppresses rDNA instability [9,10].

The interplay between rDNA stability and rRNA production by RNAPI remains unexplored. The regulation of RNAPI activity is best characterized at the level of pre-initiation complex formation. Productive RNAPI initiation depends primarily on RNAPI-Rrn3 complex which represents a small fraction of total RNAPI in exponentially growing cells [11,12]. Docking of Rrn3 to the enzyme depends on the RNAPI subunit Rpa43 [13]. Following promoter release, Rrn3 is dissociated from the RNAPI in elongation, probably by the C-terminal domain of the RNAPI subunit Rpa49 which adopts a conformation overlapping the Rrn3 binding site on the Rpa43 subunit [14,15].

Rrn3 is rapidly decayed in non-favourable growth condition such as glucose exhaustion, or artificially during TORC1 inhibition by rapamycin, resulting in disappearance of the initiation competent RNAPI-Rrn3 complexes [16]. Translational fusion of Rrn3 with Rpa43 (CARA-RNAPI) resulted in modified RNAPI activity [17]. Under non-favourable growth condition where WT RNAPI is repressed, CARA-RNAPI is able to perform several productive initiation cycles, resulting in an accumulation of rRNAs compared to wild-type RNAPI [17,18]. The fact CARA-RNAPI does not induce cell death suggests that dissociation of Rrn3 from Pol I is not a step ensuring optimal rRNA synthesis. Nevertheless, genetic studies revealed CARA-RNAPI is not viable in absence of Rpa49 suggesting that absence of Rrn3 release in cells expressing CARA-RNAPI may affect elongation processes [14,19].

We decided to assess the function of Rrn3 release from RNAPI and its consequences within the cells using CARA-RNAPI. We showed that CARA-RNAPI does not increase rRNA synthesis *in* *vivo*, but leads to a new type of rRNA processing defect: while most rRNAs are processed normally, a fraction of unprocessed 35S rRNA is accumulated. Importantly, the decay of this unprocessed 35S rRNA by the nuclear exosome is essential for cell viability. To understand why such a fraction of 35S rRNA that escapes the processing pathway is toxic for the cell, we performed a global genetic mapping using CARA-RNAPI as bait and identified an impact on rDNA stability. CARA-RNAPI showed synthetic lethality with mutants affecting rDNA organization and stability, such as monopolin mutants. The monopolin complex, containing Csm1 and Lrs4 subcomplex, is recruited to the kinetochore and rDNA, playing an important role in the accurate segregation of chromosomes [20,21]. The spatial organization of rDNA is massively disturbed in cells bearing CARA-RNAPI, and is associated with copy number variation. Fob1 deletion stabilized rDNA copy number in CARA-RNAPI mutant, but did not supress the co-lethality of the CARA-RNAPI with *csm1* deletion mutant. A drastic reduction of rDNA copy number allowed cell viability in these conditions, suggesting that the accumulation of unprocessed 35S rRNA inhibits rDNA segregation.

## Results

### Genetic interaction of CARA- RNAPI with exosome mutants

CARA-RNAPI is a deregulated RNAPI mutant characterized by the formation of an artificially Rrn3-RNAPI initiation competent complex, which is unable to release Rrn3 during elongation [17,18]. Our initial aim was to assess whether the absence of Rrn3 release could influence rRNA production, maturation or stability. To evaluate rRNA maturation and stability, we decided to compare the accumulation of rRNAs in yeast strains expressing CARA-RNAPI with or without Rrp6, a nuclear component of RNA exosome involved in maturation and degradation of various RNAs [22]. To do this, we crossed a yeast strain expressing CARA-RNAPI with a yeast strain carrying the inactivated *RRP6* gene and looked for offspring expressing CARA-RNAPI in the absence of *RRP6*. We could not recover spore with *rrp6* deletion in CARA-RNAPI background, suggesting that CARA-RNAPI is not viable in absence of Rrp6.

**Figure 1.**
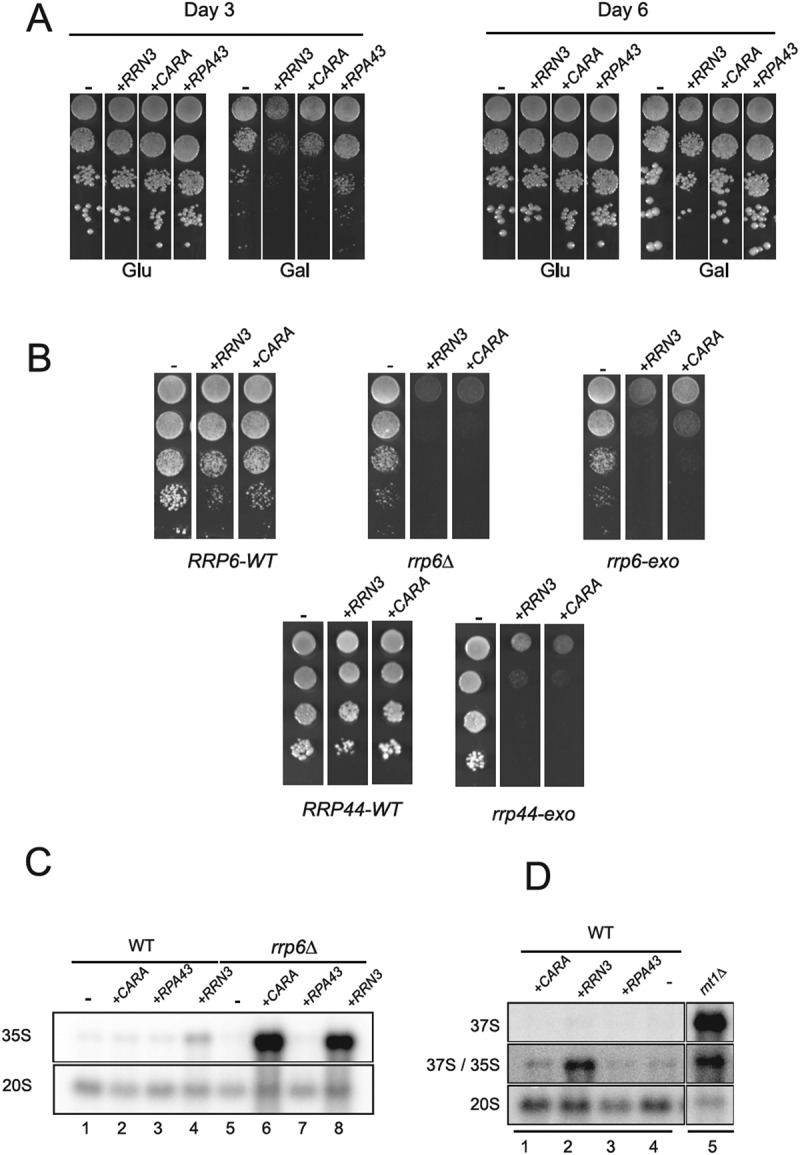
Over-expression of Rrn3 and CARA fusion is toxic in exosome mutants and leads to 35S rRNA accumulation. (A) Overexpression of Rrn3 and CARA impacts cell growth. Wild-type (WT) strains were transformed with plasmids over-expressing either Rrn3, CARA or Rpa43 under the control of galactose-dependent promoter. Ten-fold serial dilutions were seeded on both galactose containing media and glucose containing media. Growth was assessed after three and six days at 30°C. (B) The exonucleolytic activities of Rrp6 and Rrp44 are essential when CARA or Rrn3 are over-expressed. Ten-fold dilutions of WT, *rrp6Δ*, *rrp6-exo* and *rrp44-exo* strains over-expressing either Rrn3 or CARA under the control of galactose dependent promoter were seeded onto media. Growth was assessed after three days at 30°C. (C) 35S rRNA is accumulated upon overexpression of Rrn3 and CARA. Total RNAs from WT or *rrp6Δ* strains overexpressing either CARA, Rpa43 or Rrn3 were extracted, separated by gel electrophoresis and transferred to a nylon membrane. The accumulation of the 35S and 20S rRNAs was then revealed using oligonucleotide #1833 as probe (supplementary table 3). (D) Cleavage of the primary transcript at B0 site occurs upon over-expression of Rrn3 or CARA. Total RNAs from strains overexpressing CARA, Rrn3 or Rpa43 were analyzed by northern blot. 37S rRNA was specifically detected using oligonucleotide #1885 as probe.

To confirm this genetic interaction, we decided to over-express either Rpa43, Rrn3 or CARA fusion from a strong inducible promoter in wild-type and various exosome mutants (Figure 1). In wild type cells, overexpression of Rrn3 or CARA, but not Rpa43, results in a significant growth retardation visible only during the first 3 days (Figure 1A). We also overexpressed these constructs in cells with mutations affecting the exonucleolytic activities of the exosome complex components Rrp6 or Rrp44 [23,24] (Figure 1B). In contrast to the mild growth defect observed in wild-type cells, we observed strong growth defect in the mutant impacting exonucleolytic activity of Rrp6 (*rrp6-exo*), and a complete growth inhibition in absence of Rrp6. This genetic interaction suggests the involvement of Rrp6’s 3’-5’ exonuclease activity, as well as its ability to recruit an RNA substrate to the exosome’s other exonuclease, Rrp44. Since Rrp44 is essential, but its catalytic activity is not in wild-type cells, we overexpressed our constructs in a rrp44-exo mutant background. As in the case of the *rrp6-exo* background, overexpression of Rrn3 or CARA resulted in a strong growth defect in the Rrp44-exo mutant. This need for the exonuclease activity of Rrp6 and Rrp44 when Rrn3 or CARA are over-expressed suggests that toxic RNAs may accumulate within yeast cells. To investigate this further, we evaluated rRNAs accumulation by northern blot, upon over-expression of Rrn3, CARA or Rpa43 in wild-type and in absence of Rrp6 (Figure 1C). Upon 6 hours overexpression of Rrn3, and to a lesser extent of CARA, we observed an accumulation of the 35S rRNA in wild-type cells (Figure 1C, and D, lane 1–4). The accumulation of the 35S rRNA is strongly increased in absence of Rrp6 (Figure 1C, lane 6 and 8).

During rRNA synthesis, the 35S rRNA is generated by the co-transcriptional cleavage by Rnt1 (RNase III) at site B0 of the nascent transcript [25–27]. Consequently, the unprocessed 37S rRNA accumulation, corresponding to rRNA transcript from TSS to TTS, is only observed in strains defective for B0 cleavage, such as *rnt1∆* (Figure 1D, lane 5). To evaluate if Rrn3 or CARA over-expression inhibits all endonucleolytic cleavage of nascent rRNA, we used a probe detecting specifically 37S rRNA. As previously shown, we can clearly detect 37S rRNA in *rnt1∆* mutant, but not upon over-expression of Rrn3, CARA or Rpa43 (Figure 1D). Therefore, 35S rRNA accumulated upon over-expression of Rrn3 or CARA is cleaved at site B0.

We conclude that *RRN3* and CARA over-expression result in 35S rRNA accumulation, and in this context, the exonucleolytic activities of Rrp6 and Rrp44 become essential for survival.

### CARA-RNAPI is defective in rRNA processing

**Figure 2.**
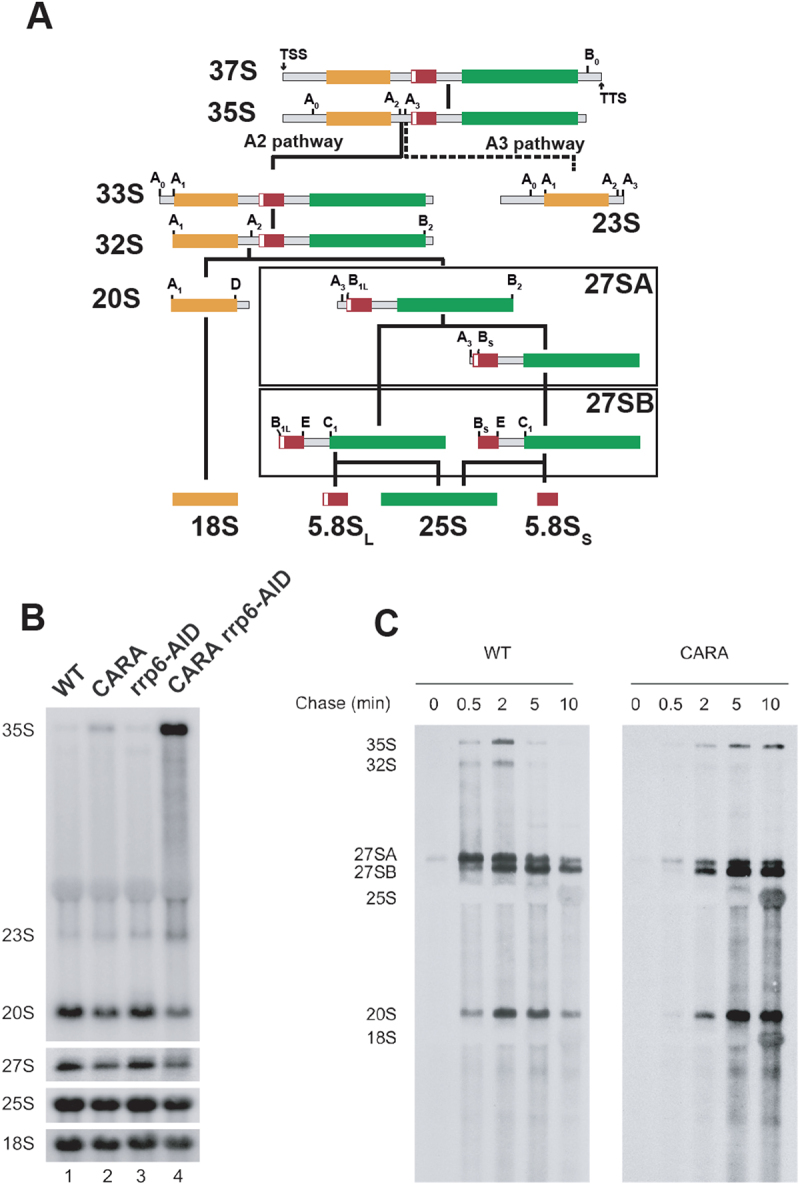
rRNA processing in CARA-RNAPI mutant. (A) Simplified pre-rRNA processing pathway in *S. cerevisiae*. Sequences corresponding to mature 18S, 5.8S and 25S rRNAs are shown in yellow, red and green, respectively. Endonucleolytic and exonucleolytic cleavages leading to the production of mature 18S, 5.8S and 25S rRNAs are detailed (A2 pathway). Inhibition of A2 pathway leads to the alternative A_3_ pathway resulting in the accumulation and/or the degradation of 23S rRNA containing particles. (B) CARA-RNAPI is defective in rRNA processing. WT, *CARA*, *rrp6-AID* and *CARA rrp6-AID* were grown to mid-log phase in glucose containing media. Cell samples were collected and total RNAs were extracted, separated by gel electrophoresis and transferred to a nylon membrane. The accumulation of the different (pre-) rRNAs was then analysed by northern blot using oligonucleotide #1833 (35S, 23S and 20S), #1830 (27S), 1829 (25S) and #892 (18S) as probe. (C) A fraction of rRNA transcripts escapes processing in CARA-RNAPI. WT and CARA strains were grown in glucose to mid-log phase. Cells were then pulse labeled with [8- [3]H] adenine for 2 min. Samples were collected 0, 0.5, 2, 5 and 10 min after the addition of an excess of cold adenine. Total RNAs were extracted from these samples, separated by gel electrophoresis and transferred to a nylon membrane.

We have shown that overexpression of CARA or Rrn3 results in an accumulation of 35S relative to wild-type, and leads to exacerbated toxicity in exosome mutants. These over-expressions were used as surrogates for the CARA-RNAPI mutant. To do so, we decided to deplete Rrp6 in CARA-RNAPI background using an auxin degron strategy. Nevertheless, we noticed that the *rrp6-AID* allele is not fully functional as shown by 5.8S + 30nt rRNA accumulation in absence of auxin (Figure S1A). We therefore decided to take advantage of this defective allele to analyse the consequences of altered Rrp6 function in cells expressing the CARA fusion. We evaluated rRNA accumulation using northern blot analysis in WT, CARA-RNAPI, *rrp6-AID* and in the double mutant combining CARA-RNAPI with *rrp6-AID*. Following synthesis by RNAPI, rRNAs are submitted to a complex process involving endo- and exo-nucleolytic cleavages, leading to the production of mature 25S, 5.8S and 18S rRNAs (Figure S2 and Figure 2A). Similarly, to the over-expression of Rrn3 or CARA, we observed an increased accumulation of 35S rRNA in CARA-RNAPI, which was strongly enhanced in *rrp6-AID* mutant background (Figure 2B). This accumulation is associated with a decreased accumulation of 20S and 27S rRNAs indicative of a rRNA processing defect. As observed with the overexpression of Rrn3 or CARA, 37S rRNA did not accumulate in the CARA background (Figure S1B).

Numerous studies of mutants affecting rRNA processing showed that the observed 35S rRNA accumulation is caused by a defect in A2-pathway (Figure 2A) This is invariably associated with a 23S rRNA accumulation, reflecting delay in the early rRNA endonucleolytic cleavages at site A0, A1, and A2, but a direct cleavage at A3 by the endonuclease MRP. The *RRP6* deletion mutant accumulates 35S and 23S rRNA, showing that A3 pathway constantly produces the 23S rRNA in wild-type cells [28,29]. In the presence of the rrp6-AID allele, no significant change in the accumulation of 35S and 23S were detected (Figure 2B). Surprisingly, in the *rrp6-AID* CARA-RNAPI double mutant, 35S rRNA accumulation was massive, whereas 23S rRNA accumulated to a much lesser extent: We measured a ratio of 35S to 23S of 9.7-fold ±2.1. Therefore, CARA-RNAPI showed a novel type of rRNA processing defect, with a 35S rRNA accumulation, little or no 23S rRNA accumulation, and a decreased 20S and 27S rRNA accumulation. We conclude that CARA-RNAPI mutant exhibits a processing defect affecting both A2 and A3 pathway of nascent rRNA (affecting endonucleolytic cleavage A0, A1, A2 and A3). Yeast strains presenting this processing defect are not viable in absence of the exonucleolytic activities of exosome.

To further clarify the processing defect detected in CARA-RNAPI strains, we conducted a kinetic analysis of rRNA processing using a [2,8- [3]H]-adenine pulse chase labelling (Figure 2C). The radioactive labelling of full-length transcripts were achieved during one minute pulse with^3^H adenine, allowing the monitoring of transcript maturation after a chase with an excess of cold adenine. Extracted RNA was analysed by gel electrophoresis to identify rRNA transcript by their size (depicted in Figure 2A). In both wild-type and CARA-RNAPI, 35S and 32S rRNAs were detected 30 seconds following the pulse, concomitantly with 27SA and 20S rRNA, both generated by the co-transcriptional cleavage of nascent rRNA at A2 site [30,31]. In wild-type cells, 35S rRNA was quickly processed into 20S and 27SA by A2 pathway, resulting in undetectable 35S rRNA after 5 to 10 min chase. In CARA-RNAPI, a fraction of 35S rRNA accumulated during the chase. The highest signal is observed after 10 min chase, indicating of a stable newly transcribed 35S rRNA accumulation. Consistent with their decreased accumulation in CARA-RNAPI, the production of 27SA and 20S rRNA was slower, but proceeded normally. Note that mature 18S and 25S rRNA are not detected due to quenching of the signal by total cellular rRNAs [31]. Therefore, CARA-RNAPI presents a highly unusual processing defect, featuring a slower, but functional rRNA production associated with the accumulation of a fraction of the 35S rRNA that remains fully unprocessed.

### rRNA processing in CARA-RNAPI is not caused by an increased rRNA production

**Figure 3.**
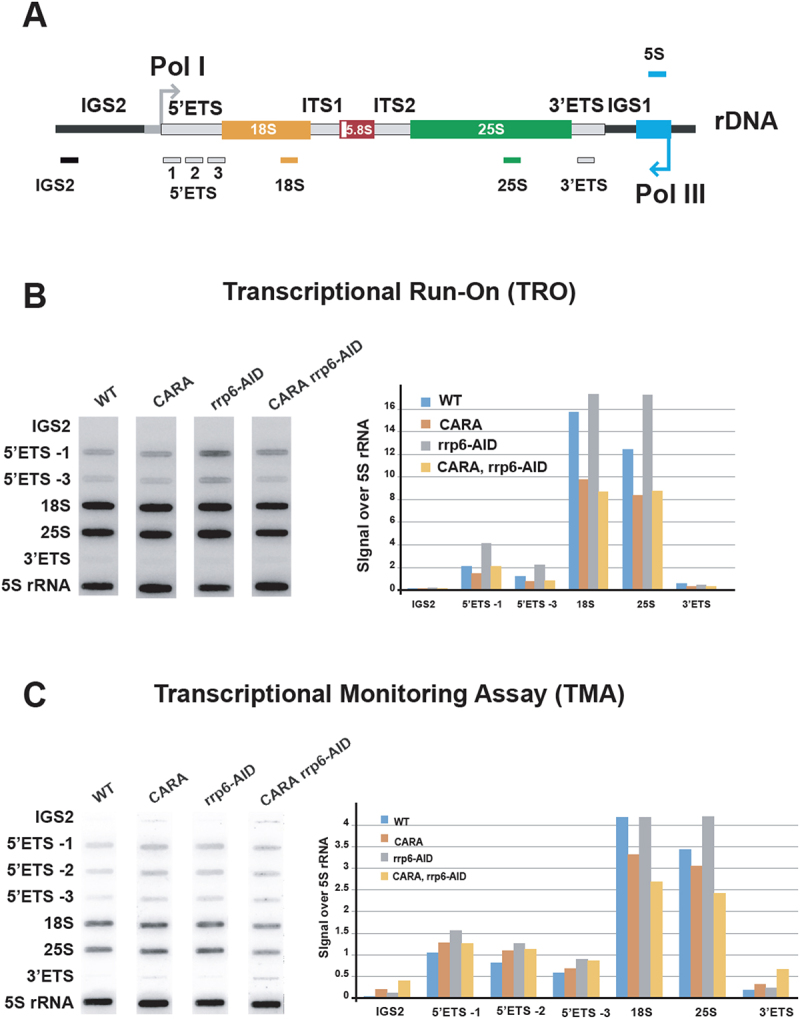
Transcriptional activity of CARA-RNAPI. (A) Yeast rDNA unit is represented, with the position of the corresponding antisense oligonucleotides used to load slot blots (see materials and methods for description). (B) High-resolution transcriptional run-on (TRO) analysis of WT, *CARA*, *rrp6-AID* and *CARA rrp6-AID* strains. Nascent transcripts were labelled, and revealed using antisens oligonucleotides immobilized on slot-blot as described in materials and methods. Results are shown in the left panel and quantifications relative to 5S signal in the right panel (arbitrary units). The full slot blot image is presented in figure S3B. (C) Pol I TMA was performed in WT, *CARA*, *rrp6-AID* and *CARA rrp6-AID* cells grown to mid-log phase in phosphate depleted YPD medium. Nascent transcripts were labelled with phosphorus-32 ([32]P]) for 40 seconds. 3’ marked newly synthesized RNAs were extracted, partially hydrolysed and revealed using slot-blots. Slot-blots are shown in the left panel and quantifications relative to 5S signal in the right panel (arbitrary units). The full slot blot image is presented in figure S3C.

The accumulation of unprocessed 35S rRNA transcript in CARA-RNAPI could be attributed to a defect in early rRNA processing, a increased transcription or a combination of both. We decided to evaluate ongoing transcription in CARA-RNAPI using high-resolution transcriptional run-on (TRO) analysis (Figure 3 and Figure S3). In TRO assay, the permeabilization of cell membranes with sarkosyl allows for the reversible blocking of elongating polymerases but also the inhibition of RNAse activities [32–35]. Transcription is next resumed in permeabilized cells in an exogenous transcription buffer in the presence of [*α*[32]P]-UTP for 5 minutes. Neosynthesized radiolabeled RNAs are then extracted and used to probe slot-blots loaded with single strand DNA fragments complementary to rDNA locus (depicted in Figure 3A and Figure S3B). Since CARA-RNAPI is constitutively active in initiation, we used two probes close to the transcription start site (TSS) to specifically investigate nascent rRNA synthesis (5’-ITS1 and 3, see material and method). Using the incorporation of [*α* [32]P]-UTP in the 5S rRNA transcribed by RNA polymerase III as an internal control, TRO revealed a two-fold decrease in rRNA transcription in CARA-RNAPI cells, irrespective of Rrp6-AID presence.

To measure *in vivo* rRNA production, and to exclude any potential bias of TRO analysis due to intrinsic inhibition of RNAses, we made use of our recently developed assay called Pol I Transcriptional Monitoring Assay (TMA) [36]. In comparison to TRO, TMA is an assay that get the advantage to monitor real *in vivo* transcriptional activity, independently of RNAse inhibition. TMA is initiated with a 40 second *in vivo* pulse labelling of all newly synthesized RNAs using incorporation of Phosphorus-32 ([32]P]) in exponentially growing cells in a rich medium depleted in phosphate. Neosynthesized, radiolabeled RNAs are next extracted, partially fragmented and used similarly to TRO to probe slot-blots loaded with single strand DNA fragments complementary to rDNA locus. As in TRO, we used 5S rRNA signal as internal control for normalization. Note that in TMA, 5S signal over 35S (including 5’-ETS, 18S, 25S and 3’-ETS) is proportionally stronger than in TRO (compare Figure 3B, and C, and Figure S3B and C). To specifically investigate rRNA produced in 5’ of rDNA gene, we used three DNA fragments complementary to sequences close to the transcription start site (TSS). TMA confirmed that CARA mutant does not exhibit an increased transcriptional activity relative to wild-type.

In conclusion, using both TRO and TMA we could not detect any increased rRNA synthesis in CARA-RNAPI mutant strains when compared to WT. We conclude that CARA-RNAPI is not a super-active form of RNAPI, but accumulates the early rRNA precursor 35S. This unusual processing defect is not caused by an increased rRNA synthesis but results exclusively from a processing defect.

### Monopolin mutants are synthetic lethal with CARA-RNAPI mutant

**Figure 4.**
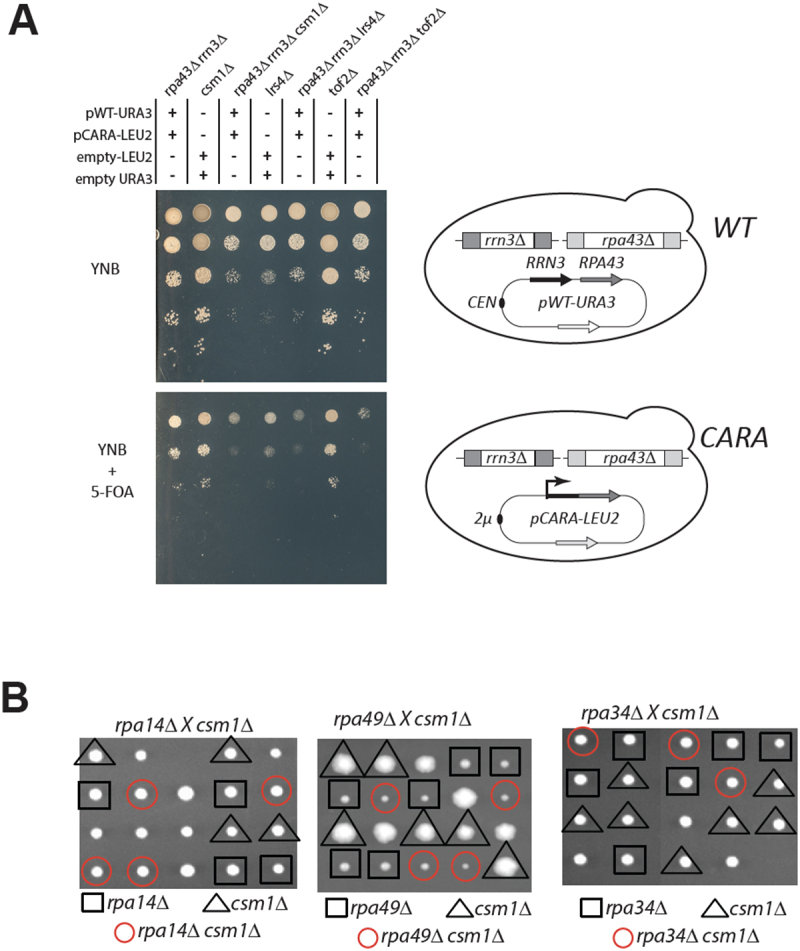
Genetic link between CARA-RNAPI and monopolin mutants. *(A) csm1*, *lrs4* and *tof2* deletion are synthetic lethal with CARA-RNAPI. Ten-fold serial dilutions of yeast bearing the indicated genotype, and construct expressing unfused *RRP43* and *RRN3* from their own promoter (pWT-URA3) and expressing the translational fusion of *RRN3* with *RPA43* (pCARA-LEU2) expressed from the strong PGK1 promoter. Empty plasmids (empty-LEU2 and empty-URA3) were used to introduce similar auxotrophic markers in all genetic background. FOA is used to counterselect cells prototroph for uracil, allowing growth of cells after loss of empty-URA3 and pWT-URA3 constructs. Viability of each mutants was evaluated by comparing plates with (FOA) or without FOA (YNB). Schematic representation of genetic background is shown on the right panel. (B) Absence of genetic interaction between *csm1* deletion and RNAPI subunit deletion *rpa14, rpa49* and *rpa34*. Strain bearing *csm1* deletion was crossed with three haploid strain bearing *rpa14*, *rpa49* and *rpa34* deletion respectively. Resulting diploids were submitted to tetrad analysis. The growth pattern of five tetrads is shown after 6 days on YPD at 30°C. Replica plating on appropriate omission media identified the genotype of individual segregants and is indicated by a shape. For each panel, single mutant is shown in black (triangle for *csm1∆*; square for RNAPI deletion). Double mutant is depicted by a red circle.

Despite the accumulation of a fraction of 35S rRNA, yeast strains carrying the CARA-RNAPI allele show no major reduction in fitness under standard culture conditions [17]. Numerous mutants affecting rRNA processing are viable when combined with *RRP6* deletion, indicating that as long as ribosomes are produced, the accumulation of rRNA precursors is not known to impair cellular viability. To identify the consequences of the non-dissociation of Rrn3 from RNAPI, leading to the accumulation of unprocessed 35S rRNA *in vivo*, we combined CARA-RNAPI with all non-essential yeast genes inactivation, using a modified version of the Genetic Interactions Mapping (GIM) method [37]. CARA-RNAPI mutant strains and an isogenic WT strain were constructed (see figure S4A). CARA-RNAPI strain was crossed to a pool of all non-essential gene deletion mutants and all possible mutants combined with CARA-RNAPI were bulk selected. Growth of the pool of CARA-RNAPI bearing deletion was next monitored and compared to the isogenic control strain on a DNA tag array. To exclude the selection of genetic interactors identified due to relative stability CEN versus 2µ plasmids, we performed a secondary screen of CARA-RNAPI against wild-type strains. From all the deletion mutants selectively depleted when combined with CARA-RNAPI mutant, we performed a candidate-based screening in which each mutant was generated individually by a plasmid shuffling assay to confirm the genetic interaction (Figure S4B). Such stringent validation steps do not aim at an exhaustive screening of the entire mutant collections, but aim to reveal robust genetic interactions. Interestingly, our results point a strong synthetic lethal (SL) phenotype with the deletion of genes coding two subunits of the monopolin complex, Csm1 and Lrs4 [21] (Figure 4A). Note that very slow-growing SL deletions with CARA-RNAPI, such as *rrp6∆* (this study) or *rpa49∆*, were not identified in our screen [14]. However, we identified some expected positive and negative genetic interactions: deletion of Rpa34, forming a heterodimer with the Rpa49 subunit, is lethal in CARA-RNAPI. We also reproduced that the deletion of Rpa14, forming a subcomplex with Rpa43 subunit, does not affect the CARA-RNAPI mutant (Figure S4C). Those genetic interaction with known RNAPI mutant showed that genetic interaction unveiled in this initial screen is specific, but may miss relevant partner.

To strengthen the genetic interaction network between CARA and the monopolin mutant identified in our screen, we then asked whether the CARA mutant genetically interacted with other mutants affecting monopolin function. The monopolin complex is recruited to the rDNA via its interaction with protein Tof2. We observed that deletion of *TOF2* is also SL with the CARA-RNAPI mutant, confirming that monopolin and its associated loader are essential in the CARA-RNAPI genetic background. It should be noted that the genetic interaction between the monopolin mutants and the CARA-RNAPI mutant is likely to be related to a defect specifically caused by CARA fusion, as *csm1Δ* is not lethal with others RNAPI mutants such as *rpa14Δ*, *rpa49Δ* or *rpa34Δ* (Figure 4B). Taken together, these results suggest that the presence of monopolin complex at the rDNA is essential in CARA-RNAPI mutant background.

### The strain bearing CARA-RNAPI exhibits rDNA organisation defect and strong copy number instability

**Figure 5.**
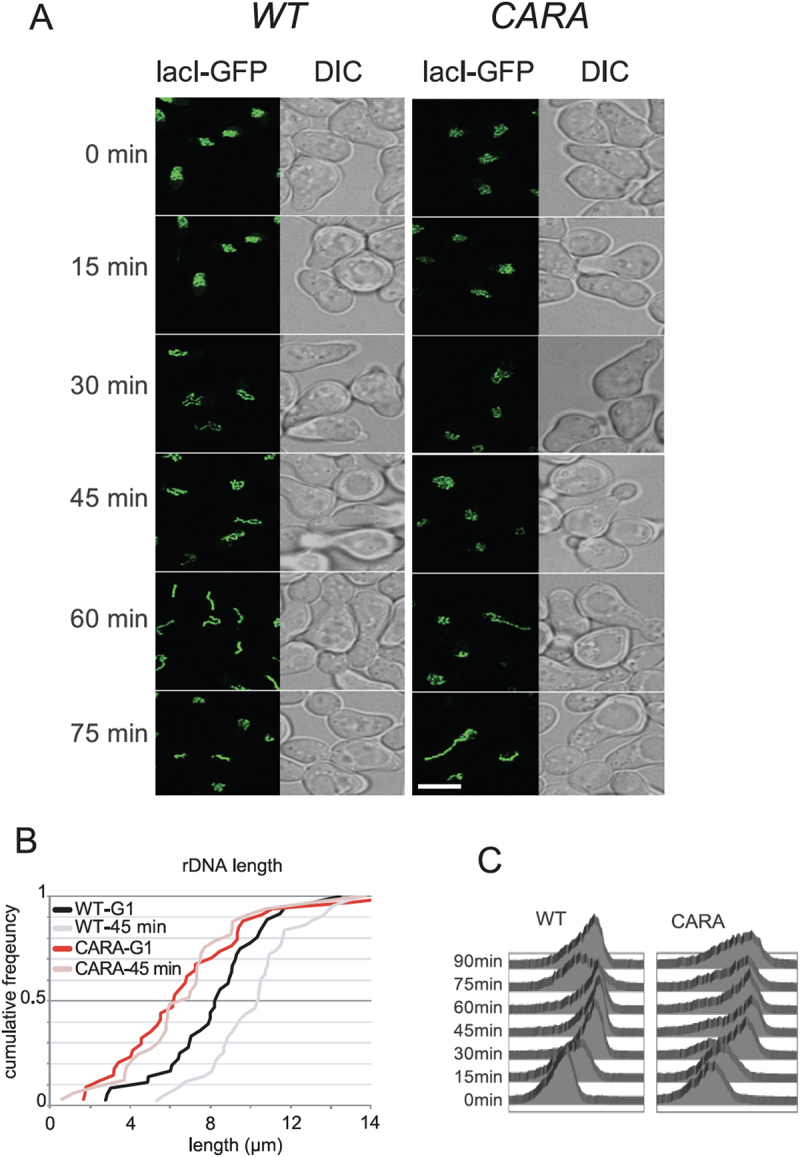
Cell cycle-dependent rDNA reorganization. (A) fluorescent imaging of rDNA structure in WT and CARA-RNAPI. Confocal imaging of WT and CARA-RNAPI strains containing a modified rDNA bearing lacO site, and labelled using LacI-GFP. Cells were arrested in G1 with alpha-factor and released synchronously in the cell cycle. Samples were taken every 15 min and processed for imaging (a) scale bar, 2 µm. (B) 3D analysis of cell cycle-dependent apparent length of rDNA. Quantitative analysis of length was quantified for cells arrested in G1 (G1), G2/M (45 min) and plotted as cumulative distribution functions using previously described image analysis pipeline [40]. (C) Flow cytometry analysis. DNA content of cells arrested in G1 with alpha-factor and released synchronously in the cell cycle were analysed using cytometry.

**Figure 6.**
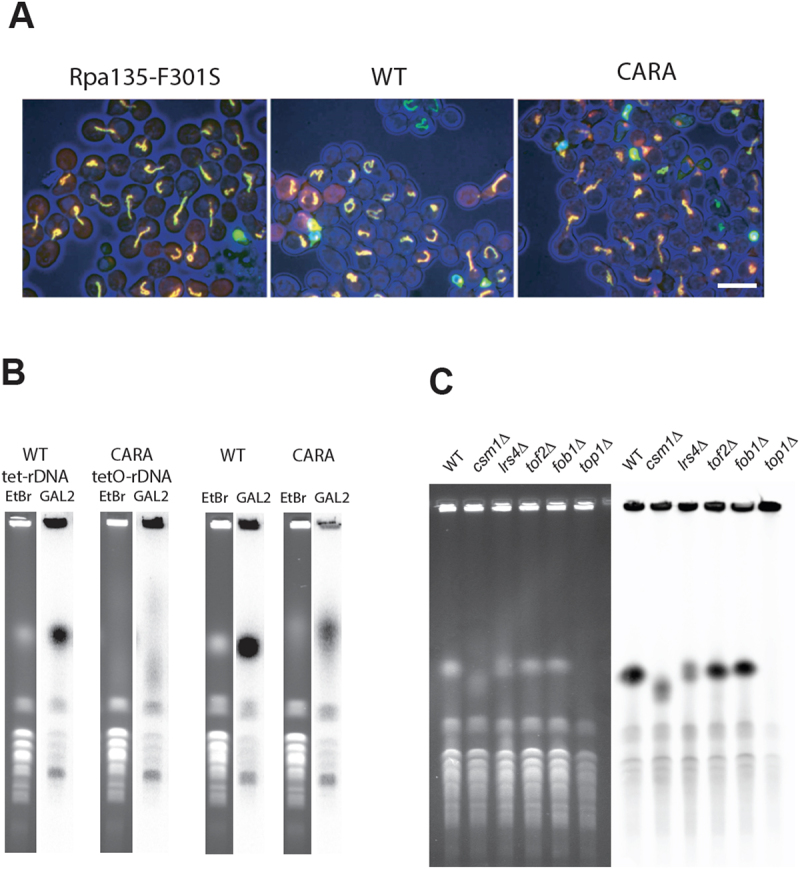
The CARA-RNAPI allele promotes rDNA instability. (A) rDNA is disorganized in G2 blocked cells in CARA-RNAPI background. Each repeat of rDNA is fluorescently labelled in vivo using lacO/lacI-GFP FROS system. G2 blocked cells were imaged in mutant RPA135-F301S (left panel), WT (middle panel) or CARA (right panel) context. Cells (blue – transmission signal) are shown with LacI-GFP signal (green) and mRFP-Nop1 staining (red). (note that mRFP-signal is not present is every cell). Scale bar : 5 µm. (B) High variability of rDNA copies number in CARA context. The size of chromosome XII was visualized using PFGE in strain bearing tetO-rDNA strain (left panels) or unlabeled rDNA strain (right panels) in WT or CARA-RNAPI context. For each strains ethidium stain gel (etbr) and southern blot of chromosome separated using PFGE are shown. A unique locus present on chromosome XII (the largest yeast chromosome) was used to detect chromosome XII independently of rDNA copies number. Note that unspecific labelling is observed on smaller chromosome. (C) Variation in rDNA size is detected in deletion mutants that are co lethal with CARA. Chromosomes from WT and *csm1∆, lrs4∆, tof2∆* deletion mutant were separated by PFGE. *fob1∆*, known to stabilize rDNA copy number, and top1∆, resulting in absence of separation of chromosome XII were used as control. Left panel: ethidium bromide stain gel. Right panel: southern blotting with rDNA specific probe (see material and methods).

The monopolin complex is known to promote the recruitment of condensin complex to rDNA, an essential step for rDNA compaction, segregation and stability during mitosis [38,39]. The co-lethality of CARA-RNAPI with monopolin could result from a synergistic defect on rDNA organization. We developed a quantitative methods to track and quantify rDNA spatial organization [40]. This method uses a modified rDNA, in which a lacO array is inserted at each rDNA unit, enabling the entire rDNA to be labelled with lacI-GFP [41]. Using strains carrying this fluorescently-tagged rDNA, we blocked cells in G1 with alpha-factor, released them into the cell cycle and imaged the rDNA pattern every 15 minutes until the completion of cell cycle. We could confirm that relative to wild-type cells, rDNA is disorganized in *csm1* and *lrs4* deletion mutant (Figure S5 and S6). We next analysed WT and CARA-RNAPI mutant using our quantitative pipeline, focusing on the apparent length of rDNA during the cell cycle (Figure 5A). In the wild type, we reproduced the massive reorganization of rDNA geometry during the cell cycle, leading to the progressive establishment of a line-like structure in G2 with nocodazole. We were able to measure a median rDNA length of 8 µm in G1, rising to 10.4 µm 45 min after exiting G1. In the CARA mutant, rDNA organization is different from that of WT from G1 to G2/M, with little detectable rDNA reorganization during the cell cycle. Measured rDNA length remains around 5 µm from G1 to G2 (Figure 5B). We also noticed a slight but reproducible delay in cell cycle after alpha-factor release in the CARA-RNAPI mutant compared to the wild-type (Figure 5C). This kinetic analysis suggests that CARA-RNAPI is defective in organizing mitotic rDNA structures. To confirm this finding, we analysed rDNA structure blocked in G2 in wild-type, CARA-RNAPI and as a control in Rpa135-F301S, a RNAPI mutant leading to an increased production of rRNAs. In cells blocked in G2 by nocodazole treatment, we were able to show that wild-type and Rpa135-F301S exhibit rDNA line-like organization (Figure 6A). Under the same conditions, the CARA-RNAPI mutant abolished the formation of this spatial organization of rDNA (Figure 6A, right panel).

During the analysis, we observed a large variation in signal intensity of rDNA in CARA-RNAPI from cell to cell, possibly indicative of some instability of the rDNA array. Therefore, we performed pulsed-field gel electrophoresis (PFGE) analysis of rDNA in CARA-RNAPI strains bearing *lacO*-rDNA or unmodified rDNA. As shown in Figure 6B, the size corresponding to chromosome XII, bearing the *lacO* rDNA array is very heterogenous in cell population expressing CARA-RNAPI compared to the WT cells. This alteration, although less dramatic, is also observed in CARA strain with unmodified rDNA (BY4741 background). As expected, deletion mutants of *csm1*, *lrs4* or to a lesser extent *tof2*, all synthetically lethal with CARA-RNAPI, also exhibit rDNA copy number variation (Figure 6C) [9,10]. Deletion of Fob1 stabilizes the number of rDNA repeats, leading to a sharper band in our assay, when compared to the WT. As previously reported, *TOP1* deletion results in undetectable Chromosome XII in PGFE, and was used here as control [42].

Taken together, these results indicate that the CARA-RNAPI mutant provokes a defect of the 3D organization of rDNA and induces a strong genetic instability of rDNA.

### rDNA Genomic instability in CARA-RNAPI depends on Fob1

**Figure 7.**
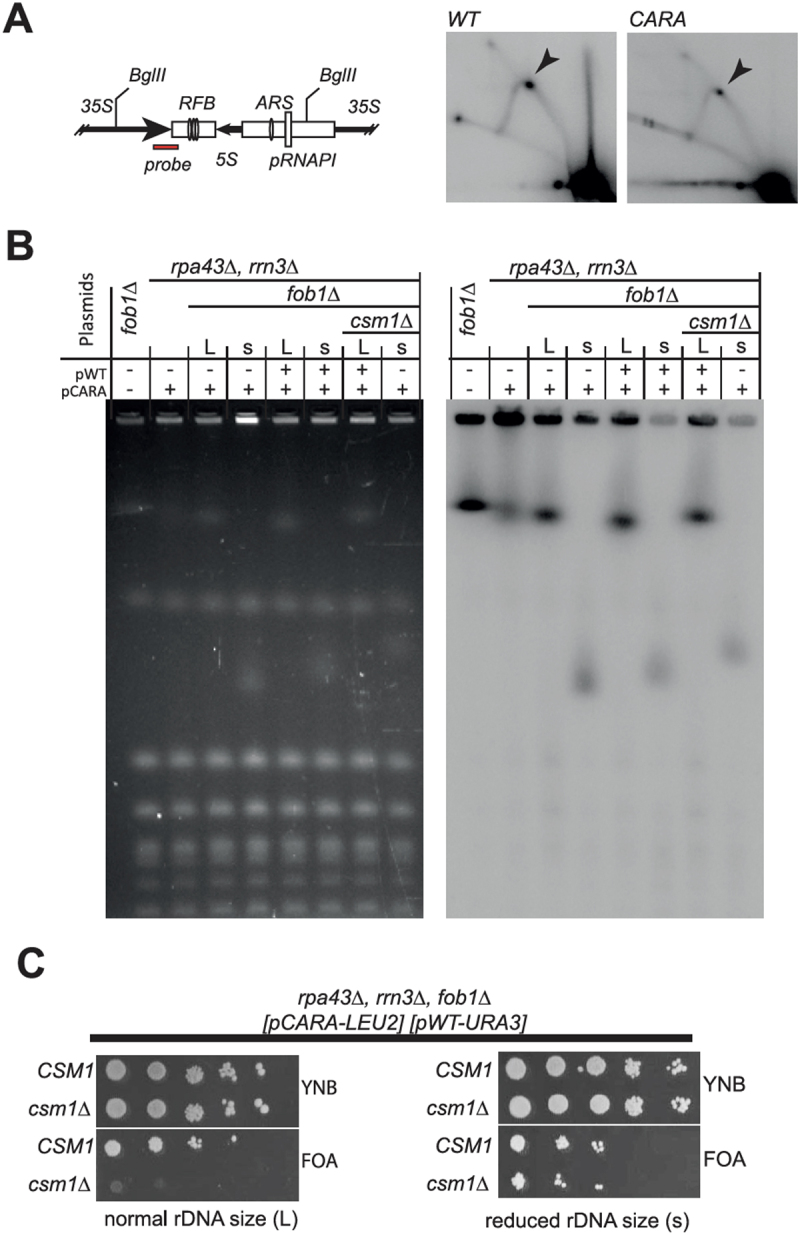
Synthetic lethality of monopolin deletion mutant *csm1* with CARA-RNAPI background is independent of replication fork barrier (RFB) activity of FobI. (A) 2D neutral-neutral agarose gel analysis of replication fork in rDNA. Left panel: schematic diagram of probe position in the *Bgl*II fragment analyzed: fork of replication barrier (RFB), 5S rDNA gene, origin of replication (ARS) and the RNAPI promoter (pRNAP1) are shown. Right panel: Southern blotting of the 2D agarose gel in WT and CARA context. The arrow indicates fork progression arrest at RFB site. (B) PFGE analysis of chromosome XII size in fob1∆ context. Fob1 deletion in CARA context allow for selection of two type of yeast: large colonies (L) bearing normal rDNA copies number and slow growing colonies (S) with reduced rDNA copy number. Southern blotting on right panel probe with an rDNA-specific fragment. Strains were complemented with either pCARA or pCARA+pWT as indicated above the wells. (C) Synthetic lethality of *csm1∆* with CARA-RNAPI is dependent on rDNA copy number. *left CSM1* is essential for growth in CARA-RNAPI *fob1∆* background bearing normal rDNA size as show by plasmid shuffling assay. *right* upon reduction of rDNA copy number, CARA-RNAPI *fob1∆ csm1∆* mutant is viable.

CARA-RNAPI has significant impacts on rDNA structure and copy number maintenance. Natural variation in rDNA copy number is under the control of the Fob1 protein, which generates a replication fork barrier (RFB) in IGS1 [6]. The activity of CARA-RNAPI may interfere with transcription termination, leading to perturbation of IGS1 chromatin status, and accumulation of non-resolved forks at RFB. We thus performed a 2D gel analysis of replication fork progression in this region. As shown in Figure 7A, we detect no change in replication fork accumulation at RFB when comparing CARA-RNAPI to wild-type. We concluded that blocked replication forks do not accumulate in the CARA-RNAPI context. The Tof2-Csm1-Lrs4 complex is recruited at the RFB by Fob1 [20]. Therefore, the deletion of *FOB1*, like monopolin deletion mutant, may be SL with CARA-RNAPI. We introduced the deletion of *FOB1* in the strain bearing CARA-RNAPI by genetic cross. After sporulation, we observed that spores bearing CARA-RNAPI and the deletion of *FOB1* were viable, but heterogeneous in growth rate, visible by small (s) or large (L) colonies size (supplementary table S1). We then checked whether this difference is induced by a difference in rDNA copy number. In both cases, the rDNA size determined by PFGE appeared as a sharp band in our assay, indicative of a stabilized rDNA copy number in each clone (Figure 7B). The rDNA size appeared drastically different, with s clone strain having a very low copy number of rDNA units whereas the size of chromosome XII is unaffected in L clones. We conclude that CARA-RNAPI *fob1∆* is viable, and that the rDNA is stabilized in the CARA-RNAPI *fob1∆* strain, indicating that the instability of rDNA in the CARA strain depends on the fork barrier activity of Fob1.

### In CARA-RNAPI mutant, rDNA size modulate monopolin complex requirement for mitosis

Deletion of *FOB1* being viable in CARA-RNAPI background, we propose that monopolin complex activity remains in absence of Fob1 protein. To test this hypothesis, we first introduced *CSM1* deletion in CARA-RNAPI *fob1∆* background associated with large rDNA copy number (L size, see supplementary table 1). To assess viability of CARA-RNAPI *fob1∆ csm1∆*, we performed plasmid shuffling assay on FOA medium (Figure 7C, left panel). The absence of growth on FOA medium shows that the triple mutant *fob1∆ csm1∆* CARA-RNAPI is lethal when the rDNA size is normal. This result confirms that monopolin complex activity remains active even in absence of Fob1 protein, and is essential in strain bearing CARA-RNAPI mutant. We next introduced *csm1∆* in mutants CARA-RNAPI - *fob1∆* bearing a reduced rDNA copy number (s size). In this genetic context, it is important to note that the triple mutant (*fob1∆ csm1∆* CARA-RNAPI) is viable, as shown by growth after plasmid shuffling assay. This indicates that a reduced rDNA size can alleviate the genetic interaction between CARA-RNAPI and *csm1* deletion. To explain why the variation in rDNA copy number generated spontaneously by the CARA-RNAPI background is not sufficient to allow growth of the *csm1∆* - CARA-RNAPI double mutant, we propose that the inactivation of Fob1 is crucial in this context for maintaining a stable propagation of a low rDNA copy number in CARA-RNAPI background, despite a clear growth defect.

We conclude that synthetic lethal interaction between monopolin deletion mutants and CARA-RNAPI allele is independent of Fob1. However, a stable reduction of rDNA length (achieved by Fob1 deletion) restore viability of CARA-RNAPI in absence of the monopolin complex.

## Discussion

In this work, we were able to define a new phenotype for an RNA polymerase I mutant: a fraction of 35S rRNAs that escapes rRNA maturation is associated with the destabilization of ribosomal DNA genes. How modifications in rRNA production rate or rRNA processing could affect rDNA stability was not previously investigated. In this study, we suggest that stable 35S rRNA directly affects rDNA stability. Furthermore, accumulation of unprocessed 35S rRNA is associated with a defect in the spatial organization of rDNA. These surprising observations reveal some interesting links with previously published studies on the dissociation of Rrn3 from transcribing Pol I and on ribosomal protein gene expression. Furthermore, a possible contribution of deregulated Pol I and/or pre-rRNA accumulation to genome stability was not documented previously, and could suggest a role for Rrn3 in genome stability in both normal and pathological contexts, such as cancer.

### Why does the absence of Rrn3 dissociation from transcribing RNAPI impact processing?

Rrn3 forms a stable complex with the monomeric form of RNAPI and is absolutely required for pre-initiation complex (PIC) formation. During promoter escape, nascent rRNA promotes the dissociation of transcribing RNAPI from promoter-bound factors by directly interacting with the Rrn7 subunit of CF and subsequently clashes with Rrn3 [15]. However, from yeast to human, Rrn3 remains associated with transcribing RNAPI in the 5’ end of transcribed genes [14,43]. In yeast, the dissociation of Rrn3 from RNAPI additionally requires the C-terminal domains of Rpa49. In CARA-RNAPI, the translational fusion prevents the dissociation of Rrn3 from elongating RNAPI. Co-transcriptional processing typically occurs on RNAPI lacking Rrn3. Unreleased Rrn3 is present near the rRNA exit channel and can cause steric hindrance, decreasing the efficiency of co-transcriptional rRNA processing factors recruitment.

### Does unprocessed 35S rRNA affect the expression of ribosomal proteins?

When rRNA production is shut down by the addition of rapamycin, ribosomal protein gene transcription is repressed by sequestering the Ifh1 transcription factor with the CURI complex [44]. In contrast, in CARA-RNAPI, ribosomal protein gene transcription remains active due to the absence of CURI complex formation [17,45]. The presence of 35S rRNA accumulation in CARA background indicates that it may have a regulatory role, by preventing the assembly of the CURI complex.

### Does unprocessed 35S rRNA affect rDNA stability?

Thanks to genetic interactions, we found evidences of rDNA instability in CARA-RNAPI. We propose that this instability is due to the accumulation of 35S rRNA in close proximity of the transcribed rDNA. During transcription, rDNA is submitted to high levels of torsional stress that is constantly released by activity of topoisomerases and the rapid eviction of nascent rRNAs. It has been proposed that nascent transcripts are physically segregated from rDNA through a phase separation mechanism [46]. Transcription by CARA-RNAPI may lead to crowding of unprocessed rRNAs close to transcribed rDNA. Accumulation of such unprocessed rRNAs onto rDNA may hinder the efficient release of physical constraints applied to this locus. This hypothesis could explain why a part of the 35S rRNA is not accessible to two endonucleases, namely Utp24, which is required for A0, A1 and A2 sites cleavages and MRP cleaving at A3 [47,48]. Additionally, this crowding could impair rDNA architecture, which persists throughout the cell cycle and prevents the spatial reorganization of rDNA required for mitosis. The defect caused by the absence of mitotic organization of rDNA can only be bypassed by reducing rDNA size.

### What is the physiological relevance of CARA-RNAPI?

CARA-RNAPI is an artificial construct designed to study the interaction of Rrn3 and RNAPI [17,18]. *RRN3* over-expression was previously used to increase the yield of RRN3/RNAPI complex purification [49,50], to increase 35S rRNA accumulation [51], and was described to be slightly toxic (see Figure 1). However, there is increasing evidence that the amount of Rrn3 is a regulator of normal and pathological growth. Investigating the Catalogue Of Somatic Mutations In Cancer, RRN3 is found mutated in about 1% of sample, and is found overexpressed mostly in breast, central nervous system, kidney, pancreas and lung cancer [52]. In cancer cells, high *RRN3* expression is associated with malignant characteristics and poor prognosis, as seen in pancreatic cancer [53]. Manipulating *RRN3* has been shown to impact mammary epithelial morphogenetic processes in breast cancer [54]. Here, we could identify a novel type of rRNA processing defect in which a fraction of 35S rRNA escapes processing, leading to rDNA instability. These findings could shed new light on those pathological roles of Rrn3 in supporting growth in cancer cells.

## Materials and methods

### Yeast culture, construction of yeast strains and plasmids

Propagation of yeast was performed using standard rich YP medium (1% yeast extract, 2% peptone) supplemented with either 2% glucose (YPD) or 2% galactose (YPG) or using minimal YNB medium (0.67% yeast nitrogen base, 0.5% (NH4)_2_SO4 and 2% glucose or galactose) supplemented with the required amino acids. Viability of mutant in CARA-RNAPI background was tested using plasmid-shuffling assays. *RPA43* and *RRN3* deletion alleles of haploid strains were complemented by the corresponding WT genes borne on *URA3*-containing plasmid pCNOD30, in presence of *LEU2*-containing plasmid expressing CARA fusion pCNOD32 (see supplementary table S2). Fluoroorotate (FOA) is toxic for URA3+ strains [55]. FOA was used to apply a strong positive selection on cells that have lost *URA3*-containing plasmid bearing WT genes, keeping CARA fusion (pCNOD32). Ten-fold serial dilutions of each tested strains were spotted on plates with (FOA) or without FOA (YNB). Growth of strains containing complementing plasmid (without FOA) is used as control.

Yeast strains used in this study are listed in supplementary table S1, plasmids in supplementary table S2 and oligonucleotides are listed in supplementary table S3. Yeast strains used in this study were derivatives of *S. cerevisiae* BY4741 and W303 background. Yeast strains were constructed by meiotic crossing, genomic integration of PCR generated DNA fragment and plasmid DNA transformation. Yeast strain bearing *RRP44* mutants were constructed in three steps, with inactivation in diploid W303 of one of the two copy of *RRP44* with PCR product obtained using oligonucleotide #1914 and #1915 using pUC19-HPH as template, followed by transformation of the resulting diploid with plasmid pCS-96 and pCS-97 [56] and selection of haploid after sporulation to generate respectively strains ySD3-6c and ySD5-2d. Rrp6 degron present in strain yNiR1-1a and FB230-3C was constructed as previously described [57]. In yeast strain yCNOD205-1a, *CSM1* deletion was obtained by single-step PCR-mediated deletion product using oligonucleotide #1677 - #1678 and pFA6-HIS3-MX6 as template. GIM screen was described previously with modification described in supplementary figure S4 [37,58].

Plasmids were constructed using cloning or Gateway technology (Invitrogen). Gateway cloning are described in supplementary table S2. Plasmid pGAL-CARA, was construct by cloning *Bam*HI-*Sal*I fragment cut from pCNOD32 into YEplac112-GAL [59] cut at same site. pGAL-RRN3 and pGAL-RPA43 plasmids were constructed by cloning PCR generated fragment using oligonucleotide #2037 - #2038 and #2039 - #2040 respectively cut *Bam*HI-*Sal*I intoYEplac112-GAL [59] cut at same site.

## RNA extractions and northern hybridizations

RNA extractions and Northern hybridizations were performed as previously described [60]. For high molecular weight RNA analysis, 4 µg of total RNA were glyoxal denatured, resolved on a 1.2% agarose gel and transferred to a nylon membrane. The sequences of oligonucleotides used to detect the RNA species are reported in supplementary table S3.

### In vivo labelling and RNA extraction and analysis

Metabolic labelling of pre-rRNA was performed as previously described [61] with the following modifications. Strains were pre-grown in synthetic glucose-containing medium lacking adenine at 30°C to an OD_600_ of 0.8 at. One-millilitre cultures were labelled with 50 µCi [8- [3]H] adenine (NET06300 PerkinElmer) for 1 min. Cells were collected by centrifugation and the pellets were frozen in liquid nitrogen. RNA was then extracted as previously described [60] and precipitated with ethanol. For high molecular weight RNA analysis, 20% of the RNA was glyoxal denatured and resolved on a 1.2% agarose gel. Low molecular weight RNAs were resolved on 8% polyacrylamide/8.3 M urea gels.

## Transcriptional run-on analysis (TRO)

TRO was performed as previously described [32,62]. Slot blots were loaded with single-stranded 80-mers DNA oligonucleotides: #1855 (IGS2), #1856 (5’ETS-1), #1858 (5’ETS-3), #1859 (18S.2), #1860 (25S.1), #1861 (3’ETS), #1863 (5S US) and #1864 (5S DS) and hybridization was performed as previously described for transcription run-on [32,62].

## Pol I TMA

Yeast cells were grown in phosphate depleted YPD medium [63] until they reached an OD600 of 0.8 at 30°C. The RNAs were labelled in vivo by incubating of 1 ml aliquots of the culture with 150 μCi [32]P]orthophosphate (p-RB-1, (54mCi/ml) Hartmann Analytic, Braunschweig, Germany) for 40 seconds. Cells were collected by centrifugation and pellets were frozen in liquid nitrogen. The RNAs were then extracted [60] and precipitated with ethanol. Slot blots were loaded and hybridized as previously described for transcription run-on with the addition of #1857 (5’ETS-2).

## 2D Gel Electrophoresis

Yeast genomic DNA was extracted from exponentially growing culture using Genomic-tip 20/G (Qiagen) as previously described [64]. After digestion of 4 µg of genomic DNA with *Bgl*II, replication intermediates were separated by neutral-neutral 2D gel electrophoresis according to Friedman K L and Brewer B J with some modification [65]. In the first dimension, gDNA was separated on 0.4% agarose, 0.5X TBE, for 60 h at 0.4 V/cm. In the second dimension, 1%, 0.5 X TBE agarose gel was run in presence of 0.3 mg/ml Ethidium Bromide at 3 V/cm for 14 hours. DNA was transferred to Hybond XL membrane (Amersham) and Southern blotting was carried out according to the manufacturer with the probe as shown Figure 7A. The probe was generated using PCR amplified using primers #317 and #322 and plasmid pNOY373 as template (Supplementary table S3) and ^32^P labelled using Prime-It RmT Random Primer Labeling Kit (Agilent #300392).

## Pulse field gel electrophoresis (PFGE)

Chromosomes were prepared from 10 O.D. of cells according to Maringele & Lydall [66]. Chromosome from 1 O.D. of cells were resolved by PFGE into CHEF-DR II System (Biorad) on 1% agarose, 0.5X TBE at 14°C (6 V/cm, initial S/time 70 s, final S/time 140 s, angle 120) for 30 hours, stain with Ethidium bromide for imaging and transfer to Hybond XL membrane (Amersham) for Southern blotting. Specific chromosome XII probes *GAL2* and rDNA were generated respectively by PCR from genomic DNA using primers #1648 and #1649 or by PstI/XhoI digestion of pNOY373 and^32^P labelled using Prime-It RmT Random Primer Labeling Kit (Agilent #300392).

## Flow cytometry

About 2,8.10^6^ cells were fixed in ethanol 70% and stored at −20°C. Cells were then pelleted, washed and incubated overnight in Tris-HCl 50 mM pH 7,5 complemented with RNase A (10 mg/mL; Sigma-Aldrich) at 37°C. Cells were pelleted, resuspended 400 ml of 1,0 mg/mL propidium iodide (Fisher, P3566) in 50 mM Tris pH 7,4, NaCl, MgCl2 and incubated for 1 h at room temperature. Flow cytometry was performed on a CyFlow ML Analyzer (Partec) and data were analysed using FloMax software.

## Microscope image acquisition

Yeast culture was diluted and exponentially growing cells were arrested in G1 by addition of α-factor (Antibody-online, ABIN399114, 1 μg/ml final) every 30 min for 2h30. After washing in cold media, cells were released synchronously and samples were taken for imaging every 15 min, for up to 90 min. Cells blocked in G2 were grown overnight on complete media, arrested in G1 with alpha-factor, and released for 2 h in complete media supplemented with nocodazole (10 m g/ml final concentration, Sigma Aldrich, M1404). Confocal microscopy was limited to 10 min after mounting and performed with a disk confocal system (Revolution Nipkow; Andor Technology) installed on an inverted microscope (IX-81; Olympus) featuring a CSU22 confocal spinning disk unit (Yokogawa Corporation of America) and an EM charge-coupled device (CCD) camera (DU 888; Andor Technology). The system was controlled using the mode ‘Revolution FAST’ of Andor Revolution IQ1 software (Andor Technology). Images were acquired using a 100× objective lens (Plan-Apochromat, 1.4 NA, oil immersion; Olympus). Single laser lines used for excitation were diode pumped solid state lasers (DPSSL) exciting GFP fluorescence at 488 nm (50 mW; Coherent) and mCherry fluorescence at 561 nm (50 mW; Cobolt jive). A bi-bandpass emission filter (Em01-R488/568–15; Semrock) allowed collection of green and red fluorescence. Pixel size was 65 nm. For 3D analysis, z stacks of 41 images with a 250-nm z step were used. Exposure time was 200 ms.

## Supplementary Material

Supplemental Material

## Data Availability

All yeast strains and plasmids are available from the corresponding authors upon request. The data supporting the findings of this study are available within the article and its supplementary materials.

## Highlights

• A fraction of 35S rRNA transcript escape processing in CARA-RNAPI

• Genetic interaction of CARA-RNAPI with RRP6, RRP44, Monopolin, ESC2 and TOF2,

• CARA-RNAPI shows Fob1 dependent rDNA copy number instability

• Chromosome segregation defect is suppressed by reduction of rDNA copy number
