# Supplementary material for: RNA polymerase I mutant affects ribosomal RNA processing and ribosomal DNA stability
Source: RNA Biol. 2024 Jul 24;21(1):1–16. doi: 10.1080/15476286.2024.2381910 (PMC11275518; doi:10.1080/15476286.2024.2381910)
Supplement: Supplemental Material [file KRNB_A_2381910_SM7368.docx]

**Supplementary table 1: Yeast strains used in this study**

| **Referred to as** | **strain** | **Genotype** | **source** |
| --- | --- | --- | --- |
|  | W303-1a | *MATa leu2-3,112 trp1-1 can1-100 ura3-1 ade2-1 his3-11,15* | ^1^ |
|  | W303-1b | *MATalpha leu2-3,112 trp1-1 can1-100 ura3-1 ade2-1 his3-11,15* | ^1^ |
|  | BMA64-1a | *MATa leu2-3,112 trp1-∆2 can1-100 ura3-1 ade2-1 his3-11,15* | ^2^ |
|  | BY4741 | *MATa his3-∆1 leu2-∆0 met15-∆0 ura3-∆0* | ^3^ |
|  | BY4742 | *MATalpha his3-∆1 leu2-∆0 lys2-∆0 ura3-∆0* | ^3^ |
| *WT*  *Figure. 1A, B, D*  *Figure 2,3,7 6B, S1* | W303-1a | *MATa leu2-3,112 trp1-1 can1-100 ura3-1 ade2-1 his3-11,15* | ^1^ |
| *rrp6∆*  *Figure 1A, 1C* | OGT47-14a | *MATa leu2-3,112 trp1-1 can1-100 ura3-1 ade2-1 his3-11,15*  *rrp6-∆::NAT-MX4* | *This study* |
| *rrp6-exo*  *Figure 1B,* | Y2068 | *MATa leu2-3,112 trp1-1 can1-100 ura3-1 ade2-1 his3-11,15*  *rrp6-Y361A* | *Euroscarf* |
| *RRP44-WT*  *Figure 1B,* | ySD3-6c | *MATalpha leu2-3,112 trp1-1 can1-100 ura3-1 ade2-1 his3-11,15*  *rrp44-∆::HPH-MX4*  *+* pCS-96 (*ARS/CEN LEU2 RRP44(WT)-szz*) | *This study* |
| *rnt1∆*  *Figure 1D, S1B* | rnt1∆ | *MATa leu2-3,112 trp1-∆2 can1-100 ura3-1 ade2-1 his3-11,15*  *rnt1-∆::-TRP1* | ^5^ |
| *rrp44-exo*  *Figure 1B,* | ySD5-2d | *MATalpha leu2-3,112 trp1-1 can1-100 ura3-1 ade2-1 his3-11,15*  *rrp44-∆::HPH-MX4*  *+* pCS-97 (*ARS/CEN LEU2 rrp44-D551N-szz*) | *This study* |
| *CARA*  *Figure 2,3, 6B, 7, S1B* | FB229-7A | *MATalpha leu2-3,112 trp1-1 can1-100 ura3-1 ade2-1 his3-11,15, rrn3∆-:: NAT-MX4, rpa43∆-::NAT-MX4*  *+ pGEN-CARA (2micron TRP1 pPGK1-CARA)* | *This study* |
| *rrp6-AID*  *Figure 2,3, S1* | yNiR1-1a | *MATa leu2-3,112 trp1-1 can1-100 ura3-1 ade2-1 his3-11,15*  *rrp6-miniAID-FLAG::KAN-MX, his3::ADH1promoter-OsTIR1-9myc::HIS3* | *This study* |
| *CARA rrp6-AID*  *Figure 2, S1* | FB230-3C | *MATa leu2-3,112 trp1-1 can1-100 ura3-1 ade2-1 his3-11,15, rrn3-∆:: NAT-MX4, rpa43-∆::NAT-MX4 rrp6-miniAID-FLAG::KAN-MX his3::ADH1promoter-OsTIR1-9myc::HIS3*  *+ pGEN-CARA (2micron TRP1 pPGK1-CARA)* | *This study* |
|  | Y24975 | *MATa/MATalpha his3-Δ1/his3-Δ1 leu2-Δ0/leu2-Δ0 LYS2/lys2-Δ0 met15-Δ0/MET15 ura3-Δ0/ura3-Δ0 RRN3/rrn3-∆::KAN-MX4* | *euroscarf* |
|  | yCNOD80-3b | *MATalpha his3-∆1 leu2-∆0 met15-∆0 ura3-∆0 rrn3-∆::KAN-MX4*  *+ pCNOD30 (ARS/CEN URA3 RRN3 RPA43)* | *This study* |
|  | yCNOD81-1a | *MATalpha his3-∆1 leu2-∆0 met15-∆0 ura3-∆0 rrn3-∆::pAlpha2-NAT-MX4 + pCNOD30 (ARS/CEN URA3 RRN3 RPA43)* | *This study* |
|  | Y21637 | *MATa/MATalpha his3-Δ1/his3-Δ1 leu2-Δ0/leu2-Δ0 LYS2/lys2-Δ0 met15-Δ0/MET15 ura3-Δ0/ura3-Δ0 RRN3/rpa43-∆::KAN-MX4* | *euroscarf* |
|  | yCNOD83-5a | *MATa his3-∆1 leu2-∆0 met15-∆0 ura3-∆0 rpa43-∆:: KAN-MX4*  *+ pCNOD30 (ARS/CEN URA3 RRN3 RPA43)* | *This study* |
|  | yCNOD85-1a | *MATa his3-∆1 leu2-∆0 met15-∆0 ura3-∆0 rpa43-∆:: HPH-MX4*  *+ pCNOD30 (ARS/CEN URA3 RRN3 RPA43)* | *This study* |
|  | yCNOD86 | *MATa/MATalpha his3-Δ1/his3-Δ1 leu2-Δ0/leu2-Δ0 LYS2/lys2-Δ0 met15-Δ0/MET15 ura3-Δ0/ura3-Δ0 RRN3/ rrn3-∆::pAlpha2-NAT-MX4 RPA43/rpa43-∆:: HPH-MX4*  *+ pCNOD30 (ARS/CEN URA3 RRN3 RPA43)* | *This study* |
| *WT*  *Figure 4 A, 7B* | yCNOD86-12b | *MATalpha his3-∆1 leu2-∆0 met15-∆0 ura3-∆0 rrn3-∆::pAlpha2-NAT-MX4 rpa43-∆:: HPH-MX4*  *+ pCNOD30 (ARS/CEN URA3 RRN3 RPA43)*  *+ pCNOD32 (2micron LEU2 pPGK1-CARA)* | *This study* |
| *CARA*  *Figure 4 A, 7B* | yCNOD86-14a | *MATalpha his3-∆1 leu2-∆0 met15-∆0 ura3-∆0 rrn3-∆::pAlpha2-NAT-MX4 rpa43-∆:: HPH-MX4*  *+ pCNOD32 (2micron LEU2 pPGK1-CARA)* | *This study* |
|  | Y15804 | *MATa his3-∆1 leu2-∆0 met15-∆0 ura3-∆0 csm1-∆::KAN-MX4* | *euroscarf* |
| *Figure 4 A, csm1* | FB16-6D | *MATa his3-∆1 leu2-∆0 met15-∆0 ura3-∆0 csm1-∆::KAN-MX4*  *+ pRS315(ARS/CEN LEU2)*  *+ pRS316(ARS/CEN URA3)* | *This study* |
| *Continued….* |  |  |  |
| **Referred to as** | **strain** | **Genotype** | **source** |
| *Figure 4 A,*  *CARA csm1* | FB16-7C | *MATalpha his3-∆1 leu2-∆0 met15-∆0 ura3-∆0 rrn3-∆::pAlpha2-NAT-MX4 rpa43 ∆:: HPH-MX4 csm1∆- ::KAN-MX4*  *+ pCNOD30 (ARS/CEN URA3 RRN3 RPA43)*  *+ pCNOD32 (2micron LEU2 pPGK1-CARA)* | *This study* |
| *lrs4∆*  *Figure 4 A, 6C* | Y04275 | *MATa his3-∆1 leu2-∆0 met15-∆0 ura3-∆0 lrs4∆- ::KAN-MX4* | *euroscarf* |
| *tof2∆*  *Figure 4 A, 6C* | Y07041 | *MATa his3-∆1 leu2-∆0 met15-∆0 ura3-∆0 tof2∆- ::KAN-MX4* | *euroscarf* |
| *top1∆*  *Figure 4 A, 6C* | Y01697 | *MATa his3-∆1 leu2-∆0 met15-∆0 ura3-∆0 top1∆- ::KAN-MX4* | *euroscarf* |
| *fob1∆*  *Figure 4 A, 6C* | Y04044 | *MATa his3-∆1 leu2-∆0 met15-∆0 ura3-∆0 fob1∆- ::KAN-MX4* | *euroscarf* |
| *rpa14∆, Figure 4 B* | Y03514 | *MATa his3-∆1 leu2-∆0 met15-∆0 ura3-∆0 rpa14∆- ::KAN-MX4* | *euroscarf* |
| *rpa49∆, Figure 4 B* | Y01196 | *MATa his3-∆1 leu2-∆0 met15-∆0 ura3-∆0 rpa49∆- ::KAN-MX4* | *euroscarf* |
| *rpa34∆, Figure 4 B* | Y01277 | *MATa his3-∆1 leu2-∆0 met15-∆0 ura3-∆0 rpa34∆- ::KAN-MX4* | *euroscarf* |
| *WT*  *Figure 5,6A* | TMY3 | *MATa leu2-3,112 trp1-1 can1-100 ura3-1 ade2-1 his3-11,15*  *rDNA-::pTM-lacO50 ::URA3, ade2::pAFS144-wtGFP::ADE2*  *+pUN100-mRFP-NOP1* | ^4^ |
| *CARA*  *Figure 5, 6A* | FB38-14D | *MATa leu2-3,112 trp1-1 can1-100 ura3-1 ade2-1 his3-11,15*  *rDNA-::pTM-lacO50 ::URA3, ade2::pAFS144-wtGFP::ADE2*  *rpa43 ∆:: HPH-MX4 rrn3-∆::KAN-MX4*  *+ pGEN-CARA (2 micron, TRP1, PGK1 promoter CARA)*  *+pUN100-mRFP-NOP1* | *This study* |
| *F301S*  *Figure 6A* | OGT44-2c | *MATa leu2-3,112 trp1-1 can1-100 ura3-1 ade2-1 his3-11,15*  *rDNA-::pTM-lacO50 ::URA3, ade2::pAFS144-wtGFP::ADE2*  *RPA135(F301S)-TAP-HIS3-MX6*  *+pUN100-mRFP-NOP1* | *This study* |
|  | yCNOD204 | *MATa/MATalpha his3-∆1/ his3-∆1 leu2-∆0/leu2-∆0 met15-∆0/ met15-∆0 ura3-∆0/ura3-∆0 rrn3-∆::pAlpha2-NAT-MX4/RRN3+ rpa43 ∆:: HPH-MX4/RPA43+ fob1∆- ::KAN-MX4 /FOB1+*  *+ pCNOD32 (2micron LEU2 pPGK1-CARA)* | *This study* |
| *Figure 7B CARA fob1 – large (L)* | yCNOD204-1a | *MATalpha his3-∆1 leu2-∆0 met15-∆0 ura3-∆0 rrn3-∆::pAlpha2-NAT-MX4 rpa43-∆:: HPH-MX4 fob1∆- ::KAN-MX4*  *+ pCNOD32 (2micron LEU2 pPGK1-CARA)*  *Normal rDNA size* | *This study* |
| *Figure 7B CARA fob1 – small (s)* | yCNOD204-3a | *MATalpha his3-∆1 leu2-∆0 met15-∆0 ura3-∆0 rrn3-∆::pAlpha2-NAT-MX4 rpa43-∆:: HPH-MX4 fob1∆- ::KAN-MX4*  *+ pCNOD32 (2micron LEU2 pPGK1-CARA)*  *small rDNA size* | *This study* |
|  | yCNOD205-1a | *MATa his3-∆1 leu2-∆0 met15-∆0 ura3-∆0 csm1-∆::HIS3-MX4* | *euroscarf* |
| *Figure 7B CARA fob1 csm1 Large (L)* | yCNOD214-1a | *MATalpha his3-∆1 leu2-∆0 met15-∆0 ura3-∆0 rrn3-∆::pAlpha2-NAT-MX4 rpa43-∆:: HPH-MX4 fob1∆- ::KAN-MX4 csm1∆- ::HIS3-MX6*  *+ pCNOD32 (2micron LEU2 pPGK1-CARA)*  *+ pCNOD30 (ARS/CEN URA3 RRN3 RPA43)*  *Normal rDNA size* | *This study* |
| *Figure 7B CARA fob1 csm1 small (s)* | yCNOD216-1a | *MATalpha his3-∆1 leu2-∆0 met15-∆0 ura3-∆0 rrn3-∆::pAlpha2-NAT-MX4 rpa43-∆:: HPH-MX4 fob1∆- ::KAN-MX4 csm1∆- ::HIS3-MX6*  *+ pCNOD32 (2micron LEU2 pPGK1-CARA)*  *+ pCNOD30 (ARS/CEN URA3 RRN3 RPA43)*  *Small rDNA size* | *This study* |
| *Figure 7C CARA fob1 csm1* | yCNOD216-1a  after FOA selection | *MATalpha his3-∆1 leu2-∆0 met15-∆0 ura3-∆0 rrn3-∆::pAlpha2-NAT-MX4 rpa43-∆:: HPH-MX4 fob1∆- ::KAN-MX4 csm1∆- ::HIS3-MX6*  *+ pCNOD32 (2micron LEU2 pPGK1-CARA)*  *Small rDNA size* | *This study* |

**Supplementary table 2: Plasmids used in this study**

| **Name** | **Description** | **Source** |
| --- | --- | --- |
| p29802 | *KAN-MX6, (PCR template)* | ^6^ |
| pUC19-HPH | *HPH-MX4,( PCR template)* | ^7^ |
| pAG25 | *NAT-MX4,( PCR template)* | ^8^ |
| pFA6-HIS3-MX6 | *HIS5 (A. gossipii)-MX6,( PCR template)* | ^9^ |
| pST1760 | *OsTIR1, tetR-VP16, TetR-Ssn6* | ^10^ |
| pST1933 | *3Xmini-AID+5Flag tag- KAN* | ^10^ |
| pGID3 | *prMFalpha2-NAT* | ^11^ |
| pASZ11 | *ARS/CEN ADE2* | ^12^ |
| pGEN-CARA | *2 micron, TRP1*, *pPGK1-CARA* | ^13^ |
| YEplac112 | *2micron, TRP1* | ^14^ |
| YEplac112-GAL | *2micron TRP1 pGAL* | ^15^ |
| pGAL-CARA | *2micron TRP1 pGAL CARA* | This study |
| pGAL-RPA43 | *2micron TRP1 pGAL RPA43* | This study |
| pGAL-RRN3 | *2micron TRP1 pGAL RRN3* | This study |
| Empty-URA3 | *ARS/CEN URA3 (pRS316)* | ^16^ |
| Empty-LEU2 | *ARS/CEN LEU2 (pRS315)* | ^16^ |
| pCS-96 | *ARS/CEN LEU2 RRP44-szz (pRS315)* | ^17^ |
| pCS-97 | *ARS/CEN LEU2 rrp44-D551N-szz (pRS315)* | ^17^ |
| pDONR221 | *Gateway Cloning Vector* | Invitrogen |
| pDONR221-P1P5r | *Gateway Cloning Vector* | Invitrogen |
| pDONR221-P5P2 | *Gateway Cloning Vector* | Invitrogen |
| pRS425-PGK | *2micron, pPGK1, LEU2*  *Gateway destination vector* | ^18^ |
| pFL36CII | *ARS/CEN, LEU2*  *Gateway destination vector* | ^18^ |
| pCNOD31 | *ARS/CEN URA3*  *Gateway derivative of pRS316 bearing Insertion of RCF cassette between HindIII and KpnI site.* | This study |
| pCNOD24 | *BP cloning of PCR generated fragment bearing RRN3 (oligo 680-682) using yeast genomic DNA as template into pDONR221-P1P5r. The resulting insert is flancked by attL1 and attR5 sites.* | This study |
| pCNOD25 | *BP cloning of PCR generated fragment bearing RRN3 (oligo 689-681) using yeast genomic DNA as template into pDONR221. The resulting insert is flancked by attL1 and attL2 sites.* | This study |
| pCNOD26 | *BP cloning of PCR generated fragment bearing RPA43 (oligo 677-678) using yeast genomic DNA as template into pDONR221. The resulting insert is flancked by attL1 and attL2 sites.* | This study |
| pCNOD27 | *BP cloning of PCR generated fragment bearing RPA43 (oligo 678-679) using yeast genomic DNA as template into pDONR221-P1P5r. The resulting insert is flancked by attL1 and attR5 sites* | This study |
| pCNOD28 | *ARS/CEN URA3 RPA43*  *LR cloning of entry clone pCNOD26 into destination vector pCNOD31* | This study |
| pCNOD29 | *ARS/CEN URA3 RRN3*  *LR cloning of entry clone pCNOD25 into destination vector pCNOD31* | This study |
| pCNOD30 | *ARS/CEN URA3 RRN3 RPA43*  *LR cloning of two entry clone pCNOD24 and pCNOD27 into destination vector pCNOD31* | This study |
| pCNOD32 | *2micron LEU2 pPGK1-CARA*  *Constrution by gap repair using pGEN-CARA (fragment AflIII) and pRS425-pPGK digest NheI/BglII into BY4741 yeast strain.* | This study |
| pCNOD33 | *ARS/CEN LEU2 RRN3*  *LR cloning of entry clone pCNOD25 into destination vector pFL36CII* | This study |
| pCNOD34 | *ARS/CEN LEU2 RPA43*  *LR cloning of entry clone pCNOD26 into destination vector pFL36CII* | This study |
| pUN100-mRFP-NOP1 | *ARS/CEN LEU2 mRFP-NOP1* | ^19^ |
| pNOY373 | *2micron, LEU2, rDNA* | ^20^ |

**Supplementary table 3: Oligonucleotides used in this study**

| Number | Used for | 5’-3’ Sequence |
| --- | --- | --- |
| 317 | PFGE probe, PCR rDNA, | AAAGAAGACCCTGTTGAGCTTGA |
| 322 | PFGE probe, PCR rDNA | AACAAATCAGACAACAAAGGCTTAATC |
| 677 | Gateway cloning of *RPA43* | GGGGACAAGTTTGTACAAAAAAGCAGGCTTTTTCTTTCTGGCTTTGATTATAATCG |
| 678 | Gateway cloning of *RPA43* | GGGGACCACTTTGTACAAGAAAGCTGGGTTCTATACTAGCAAGCCTCGAATC |
| 679 | Gateway cloning of *RPA43* | GGGGACAACTTTGTATACAAAAGTTGTTTTCTTTCTGGCTTTGATTATAATCG |
| 680 | Gateway cloning of *RRN3* | GGGGACAAGTTTGTACAAAAAAGCAGGCTACTTAGTGAATACATTATCAACTAG |
| 681 | Gateway cloning of *RRN3* | GGGGACCACTTTGTACAAGAAAGCTGGGTTGCTCTTGTATACCTTCAAGTTT |
| 682 | Gateway cloning of *RRN3* | GGGGACAACTTTTGTATACAAAGTTGTGCTCTTGTATACCTTCAAGTTT |
| 892 | northern probe 008, detection of 18S rRNA | CATGGCTTAATCTTTGAGAC |
| 1648 | PFGE probe, PCR *GAL2* | CTGGAAGAAAGTCCAGGCAAGTACC |
| 1649 | PFGE probe, PCR *GAL2* | TGTTTTACCTTGGAAATCTGAAGGC |
| 1677 | Deletion of *CSM1* | CAGGTATGCAAAGGAATAATCGGAATTTTATGTGTAGATATATAGACATTATGTAGCAGCTCGATGAATTCGAGCTCGTT |
| 1678 | Deletion of *CSM1* | ATAGATAAACAGGAACAATAAAAAAAAGAAAGCGAAACAAGACAATCCAACGTGCGCAAAGGTCGACGGATCCCCGGGTT |
| 1829 | northern probe 007, detection of 25S rRNA | CTCCGCTTATTGATATGC |
| 1830 | northern probe 020, detection of 35S, 33/32S, 27S, 7S and 5.8S+30 rRNAs | TGAGAAGGAAATGACGCT |
| 1833 | northern probe 004 ITS1 between D and A2 | CGGTTTTAATTGTCCTA |
| 1855 | Run-ON and TMA  *IGS2* | AAATGGCCTATCGGAATACATTTTCTACATCCTAACTACTATAAAACAACCTTTAGACTTACGTTTGCTACTCTCATGGT |
| 1856 | Run-ON and TMA  *5'-ETS-1* | TACAAAAACATAACGAACGACAAGCCTACTCGAATTCGTTTCCAAACTCTTTTCGAACTTGTCTTCAACTGCTTTCGCAT |
| 1857 | Run-ON and TMA  *5'-ETS-2* | tgcgaccggctattcaacaaggcattcccccaagtttgaattctttgaaatagattgctattagctagtaatccaccaaa |
| 1858 | Run-ON and TMA  *5'-ETS-3* | TATCTTAAAAGAAGAAGCAACAAGCAGTAAAAAAGAAAGAAACCGAAATCTCTTTTTTTTTTTCCCACCTATTCCCTCTT |
| 1859 | Run-ON and TMA  *18S* | ggaattcctcgttgaagagcaataattacaatgctctatccccagcacgacggagtttcacaagattaccaagacctctc |
| 1860 | Run-ON and TMA  *25S* | gtgctggcctcttccagccataagaccccatctccggataaaccaattccggggtgataagctgttaagaagaaaagata |
| 1861 | Run-ON and TMA  *3'-ETS* | gtaaatggtacactcttacacactatcatcctcatcgtatattataatagatatatacaatacatgtttttacccggatc |
| 1863 | Run-ON and TMA  *5S US* | cagcttaactacagttgatcggacgggaaacggtgctttctggtagatatggccgcaaccgatagtttaacggaaacgca |
| 1864 | Run-ON and TMA  *5S DS* | aaaaaaaaaaaagaaataaagattgcagcacctgagtttcgcgtatggtcacccactacactactcggtcaggctcttac |
| 1885 | 37S probe | ACAAATAAAATTTATAGAGACTTGTTCAGTCTACTTCTCTCTAAACTAGGCCCCGGCTCCTGCCAGTACCCACTTAGAAA |
| 1914 | Deletion of *RRP44* | TTCTTAGAGATACATT GTGAGGGACCCATTGA TCAAACGAGTTTTATT TATCATACTTGCATCAT ACAGGCCAAAACAAC TGTAAAACGACGGCC AGT |
| 1915 | Deletion of *RRP44* | CAGGAAACAGCTATGACCATGTGACTACTTT ACGATGTGTTTTATATA TGAGTTATGAATTCCT TTTCGTTTTTATATCCT GATACTGAAGCATCTT CCAT |
| 2037 | Cloning *RRN3* | ccgggcgggtcgacttagtcatccgacccatcac |
| 2038 | Cloning *RRN3* | gggcgggggggatccatgatggcttttgagaatac |
| 2039 | Cloning *RPA43* | ccgggcgggtcgacctaatcactatcactcgatt |
| 2040 | Cloning *RPA43* | gggcgggggggatccatgtcacaagtaaaaagagccaatgagaa |
| 2100 | northern probe 017, detection of 5.8S, 5.8S+30 and 7S RNAs | GCGTTGTTCATCGATGC |
| 2101 | northern probe 041, detection of 5S rRNA | CTACTCGGTCAGGCTC |

Bibliography

1. Thomas BJ, Rothstein R. Elevated recombination rates in transcriptionally active DNA. Cell 1989; 56:619–30.

2. Baudin-Baillieu A, Guillemet E, Cullin C, Lacroute F. Construction of a yeast strain deleted for the TRP1 promoter and coding region that enhances the efficiency of the polymerase chain reaction-disruption method. Yeast 1997; 13:353–6.

3. Brachmann CB, Davies A, Cost GJ, Caputo E, Li J, Hieter P, Boeke JD. Designer deletion strains derived from Saccharomyces cerevisiae S288C: a useful set of strains and plasmids for PCR-mediated gene disruption and other applications. Yeast 1998; 14:115–32.

4. Miyazaki T, Kobayashi T. Visualization of the dynamic behavior of ribosomal RNA gene repeats in living yeast cells. Genes Cells 2011; 16:491–502.

5. Chanfreau G, Rotondo G, Legrain P, Jacquier A. Processing of a dicistronic small nucleolar RNA precursor by the RNA endonuclease Rnt1. EMBO J 1998; 17:3726–37.

6. Cabal GG, Genovesio A, Rodriguez-Navarro S, Zimmer C, Gadal O, Lesne A, Buc H, Feuerbach-Fournier F, Olivo-Marin J-C, Hurt EC, et al. SAGA interacting factors confine sub-diffusion of transcribed genes to the nuclear envelope. Nature 2006; 441:770–3.

7. Berger AB, Decourty L, Badis G, Nehrbass U, Jacquier A, Gadal O. Hmo1 is required for TOR-dependent regulation of ribosomal protein gene transcription. Mol Cell Biol 2007; 27:8015–26.

8. Goldstein AL, McCusker JH. Three new dominant drug resistance cassettes for gene disruption in Saccharomyces cerevisiae. Yeast 1999; 15:1541–53.

9. Longtine MS, McKenzie A, Demarini DJ, Shah NG, Wach A, Brachat A, Philippsen P, Pringle JR. Additional modules for versatile and economical PCR-based gene deletion and modification in Saccharomyces cerevisiae. Yeast 1998; 14:953–61.

10. Tanaka S, Miyazawa-Onami M, Iida T, Araki H. iAID: an improved auxin-inducible degron system for the construction of a “tight” conditional mutant in the budding yeast Saccharomyces cerevisiae. Yeast 2015; 32:567–81.

11. Decourty L, Saveanu C, Zemam K, Hantraye F, Frachon E, Rousselle J-C, Fromont-Racine M, Jacquier A. Linking functionally related genes by sensitive and quantitative characterization of genetic interaction profiles. Proc Natl Acad Sci USA 2008; 105:5821–6.

12. Stotz A, Linder P. The ADE2 gene from Saccharomyces cerevisiae: sequence and new vectors. Gene 1990; 95:91–8.

13. Laferté A, Favry E, Sentenac A, Riva M, Carles C, Chédin S. The transcriptional activity of RNA polymerase I is a key determinant for the level of all ribosome components. Genes Dev 2006; 20:2030–40.

14. Gietz RD, Sugino A. New yeast-Escherichia coli shuttle vectors constructed with in vitro mutagenized yeast genes lacking six-base pair restriction sites. Gene 1988; 74:527–34.

15. Roig MB, Löwe J, Chan K-L, Beckouët F, Metson J, Nasmyth K. Structure and function of cohesin’s Scc3/SA regulatory subunit. FEBS Lett 2014; 588:3692–702.

16. Sikorski RS, Hieter P. A system of shuttle vectors and yeast host strains designed for efficient manipulation of DNA in Saccharomyces cerevisiae. Genetics 1989; 122:19–27.

17. Schneider C, Anderson JT, Tollervey D. The exosome subunit Rrp44 plays a direct role in RNA substrate recognition. Mol Cell 2007; 27:324–31.

18. Albert B, Léger-Silvestre I, Normand C, Ostermaier MK, Pérez-Fernández J, Panov KI, Zomerdijk JCBM, Schultz P, Gadal O. RNA polymerase I-specific subunits promote polymerase clustering to enhance the rRNA gene transcription cycle. J Cell Biol 2011; 192:277–93.

19. Albert B, Colleran C, Léger-Silvestre I, Berger AB, Dez C, Normand C, Perez-Fernandez J, McStay B, Gadal O. Structure-function analysis of Hmo1 unveils an ancestral organization of HMG-Box factors involved in ribosomal DNA transcription from yeast to human. Nucleic Acids Res 2013; 41:10135–49.

20. Wai HH, Vu L, Oakes M, Nomura M. Complete deletion of yeast chromosomal rDNA repeats and integration of a new rDNA repeat: use of rDNA deletion strains for functional analysis of rDNA promoter elements in vivo. Nucleic Acids Res 2000; 28:3524–34.
